# Supplementary material for: The coevolution of lifespan and reversible plasticity
Source: Nat Commun. 2019 Feb 1;10:538. doi: 10.1038/s41467-019-08502-9 (PMC6358619; doi:10.1038/s41467-019-08502-9)
Supplement: Supplementary file 1 — Supplementary Information [file 41467_2019_8502_MOESM1_ESM.pdf]

Supplementary Information

The coevolution of lifespan and reversible plasticity

Ratikainen and Kokko

TABLE OF CONTENTS

Supplementary Figure 1: Senescence 2
Supplementary Figure 2: Coevolutionary trajectories 3
Supplementary Figure 3: Evolutionary trajectories 4
Supplementary Note 1: Changing costs of plasticity 5
Supplementary Note 2: Costs of reproduction 7
Supplementary Note 3: Phenotypic mismatch affects adult mortality only 8
Supplementary Note 4: Phenotypic mismatch affect fecundity only 9
Supplementary Note 5: ARMA modelled environment 10

SUPPLEMENTARY FIGURE 1: SENESCENCE

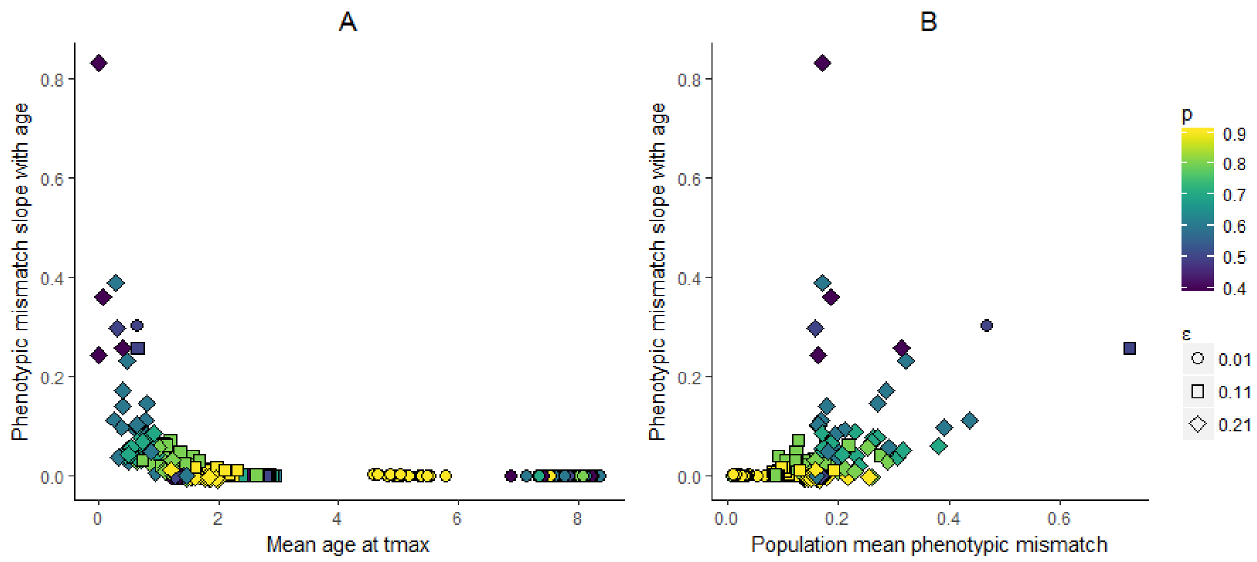

Supplementary Figure 1. Within population relationship between increase in phenotypic mismatch with age (positive slope, on the y-axis). A) showing how the "senescence" (increase in phenotypic mismatch with age) is related to lifespan. There is more "senescence" in populations that generally do not live very long. **B)** Showing how "senescence" is related to the general level of phenotypic mismatch in the population. It is a few populations with strong environmental autocorrelation (dark blue) and intermediate levels of mean phenotypic mismatch in the population that has the strongest increase in mismatch with age. ε is the updating error, and p is the autocorrelation of the environment.

SUPPLEMENTARY FIGURE 2: COEVOLUTIONARY TRAJECTORIES

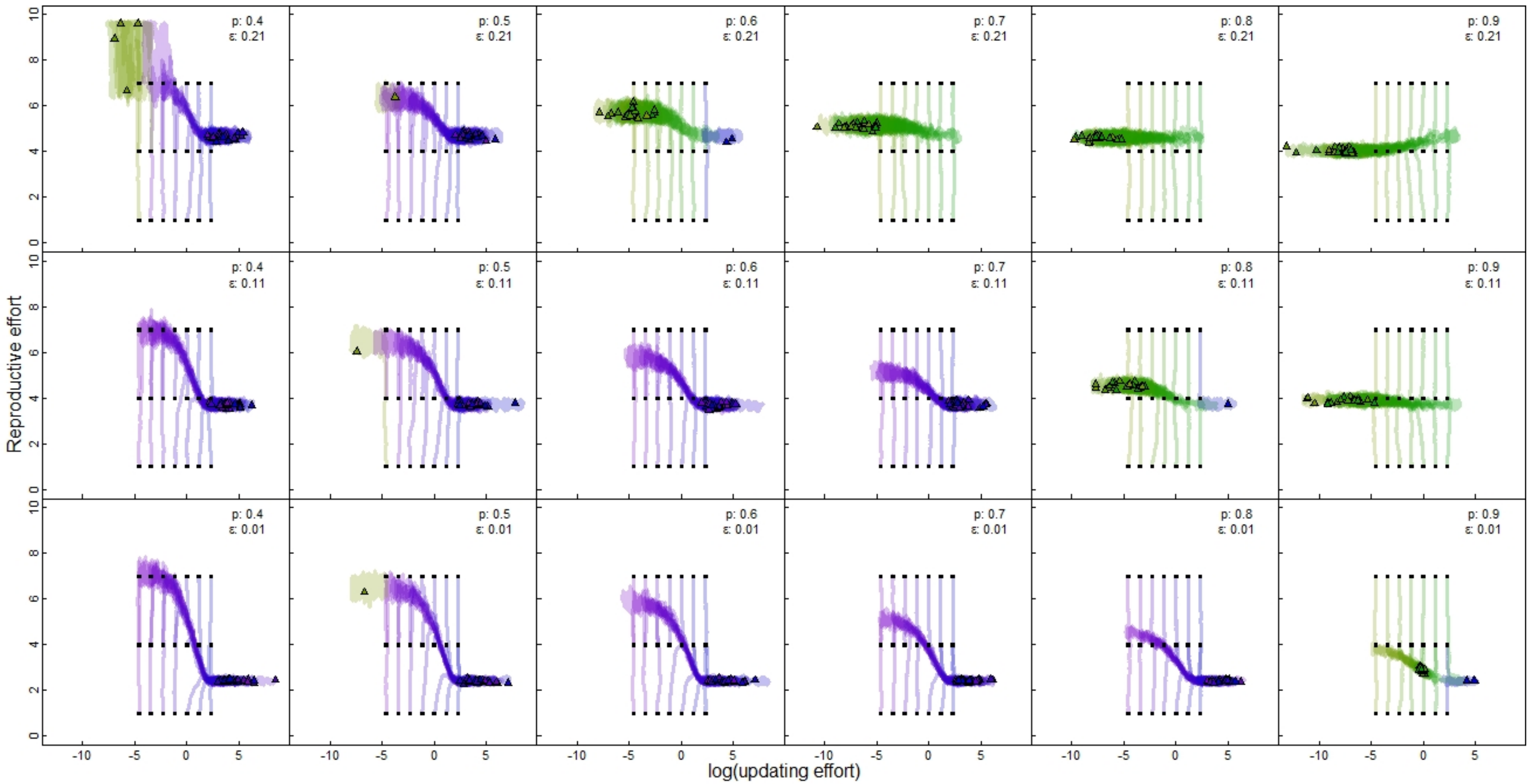

Supplementary Figure 2. Evolutionary trajectories for mean reproductive effort, r, against mean updating effort, measured as log(*u*) for all different combinations of environmental autocorrelation and updating error. Triangles indicate trait values at end of the simulation (t=50.000). ε is the updating error, and p is the autocorrelation of the environment. The colour of the line and endpoint indicate if the final updating effort is >0.5 (blue) or <0.5 (green). In most scenarios all populations end up at the same stategy (either blue or green lines and endpoints), but for some populations, evolved strategy depends on the starting gene values in the population.

SUPPLEMENTARY FIGURE 3: EVOLUTIONARY TRAJECTORIES

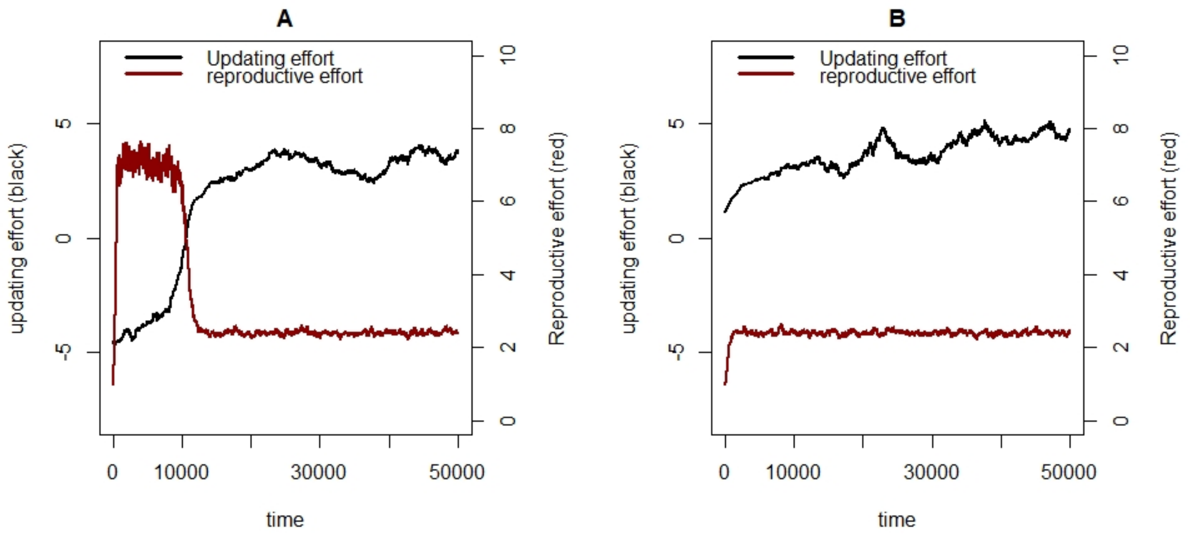

Supplementary Figure 3. Evolution of updating effort (black) and reproductive effort (red) over time. p=0.4, ε=0.01, r_{init}=1. Initial values of updating effort (**A**: 0.01; **B**: 3.1623) affect the evolutionary trajectory. This example clearly show how reproductive effort first evolves fast, then updating effort evolves more slowly and updating effort then evolves back to very low values. For some runs of the simulation, this process takes a very long time and the transient equilibrium where updating is low and reproductive effort is high will last for a long time.

SUPPLEMENTARY NOTE 1: CHANGING COSTS OF PLASTICITY

Increasing or decreasing the cost of plasticity has very little qualitative effect on the model results. Low costs of updating (κ=0.2) generally leads to more updating in most scenarios (Supplementary Figure 4), while increasing the costs (κ=0.6) leads to less updating and plasticity (Supplementary Figure 5), but the overall pattern is similar to the main results.

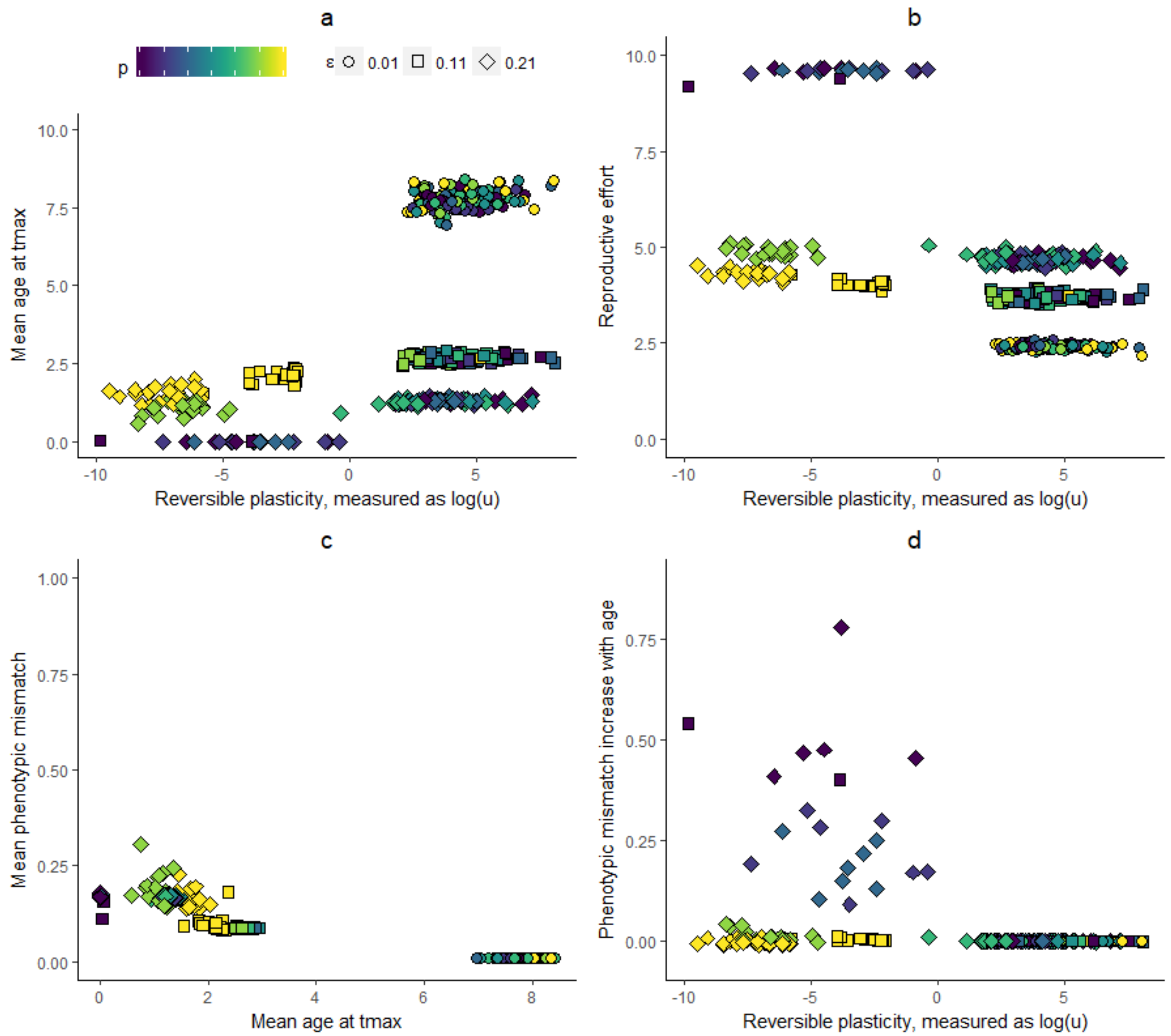

Supplementary Figure 4 Relationships between populations mean trait values at the end of all simulations with low costs of updating (κ=0.2). A) Mean age of individuals — a measure of lifespan — measured at the end of the simulation (t_{max}); long lifespan is only found at high plasticity levels. **B)** Mean evolved reproductive effort (gene value) covaries negatively with plasticity, measured as updating effort, u. **C)** Mean population-wide mismatch against mean age of individuals measured at t_{max}. **D)** The regression slope between individual mismatch and individual age, measured at t_{max}, with positive values indicating that older individuals are more mismatched to the current environment. Frequent enough plasticity can prevent this type of senescence. Symbol shapes indicate updating error (epsilon); colour (from dark blue to yellow) indicates increasing environmental autocorrelation (p) ranging from 0.4 to 0.9.

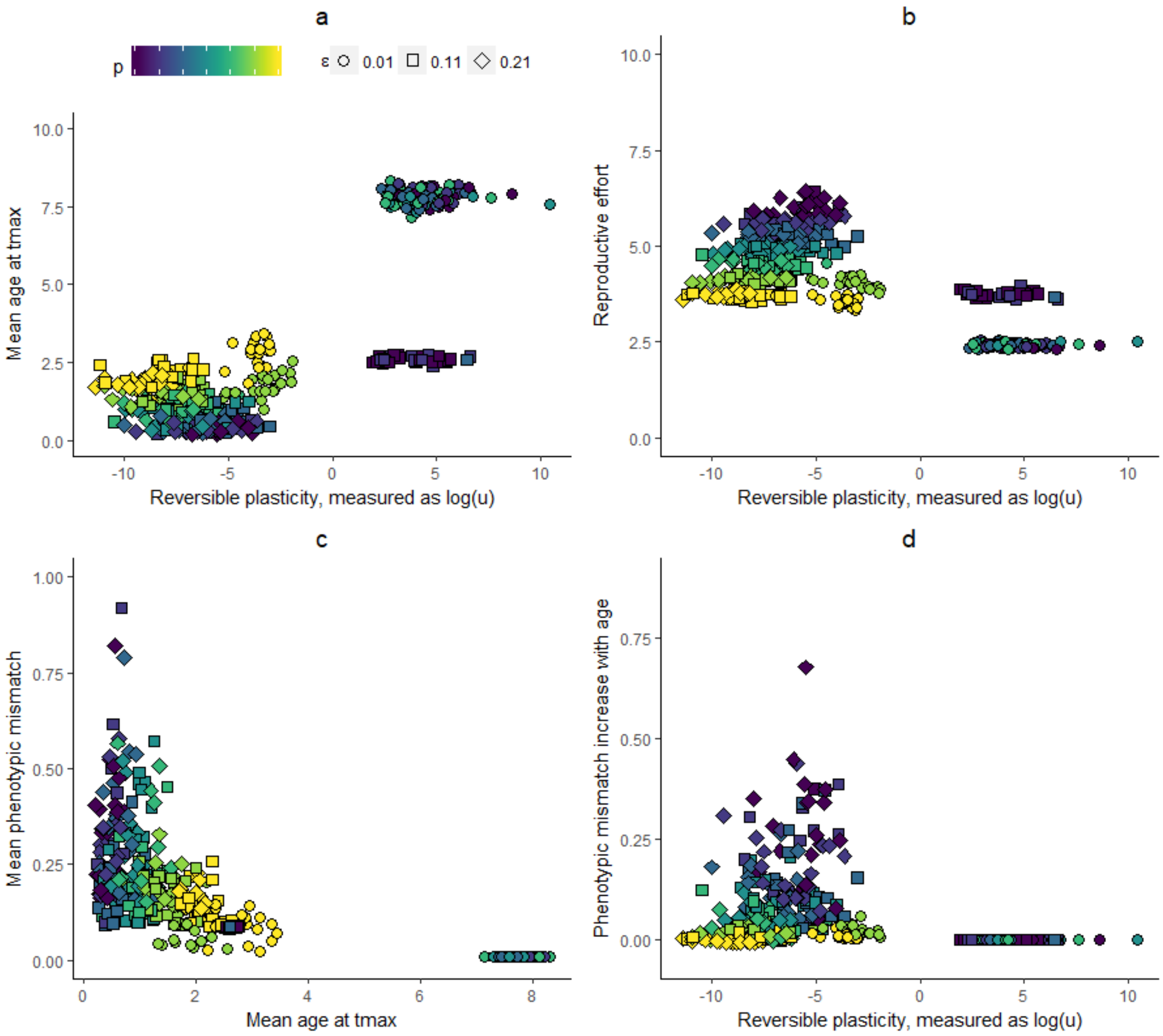

Supplementary Figure 5. Relationships between populations mean trait values at the end of all simulations with high costs of updating (κ=0.6). A) Mean age of individuals — a measure of lifespan — measured at the end of the simulation (t_{max}); long lifespan is only found at high plasticity levels. **B)** Mean evolved reproductive effort (gene value) covaries negatively with plasticity, measured as updating effort, *u*. **C)** Mean population-wide mismatch against mean age of individuals measured at t_{max}. **D)** The regression slope between individual mismatch and individual age, measured at t_{max}, with positive values indicating that older individuals are more mismatched to the current environment. Frequent enough plasticity can prevent this type of senescence. Symbol shapes indicate updating error (ε); colour (from dark blue to yellow) indicates increasing environmental autocorrelation (*p*) ranging from 0.4 to 0.9.

Supplementary Note 2: Costs of reproduction

Increasing the cost of reproductive effort from 0.01 to *ρ*=0.05, we find two distinct reproductive strategies evolving. One of those strategies involves maximum investment in reproduction and thus hardly any individuals survive more than one time step, and investment in updating and plasticity is selectively neutral. For the populations where reproductive effort does not evolve to be very high, there is still a negative relationship between reproductive effort and reversible plasticity, and a positive relationship between plasticity and lifespan, but this is driven mainly by the populations with very little updating error.

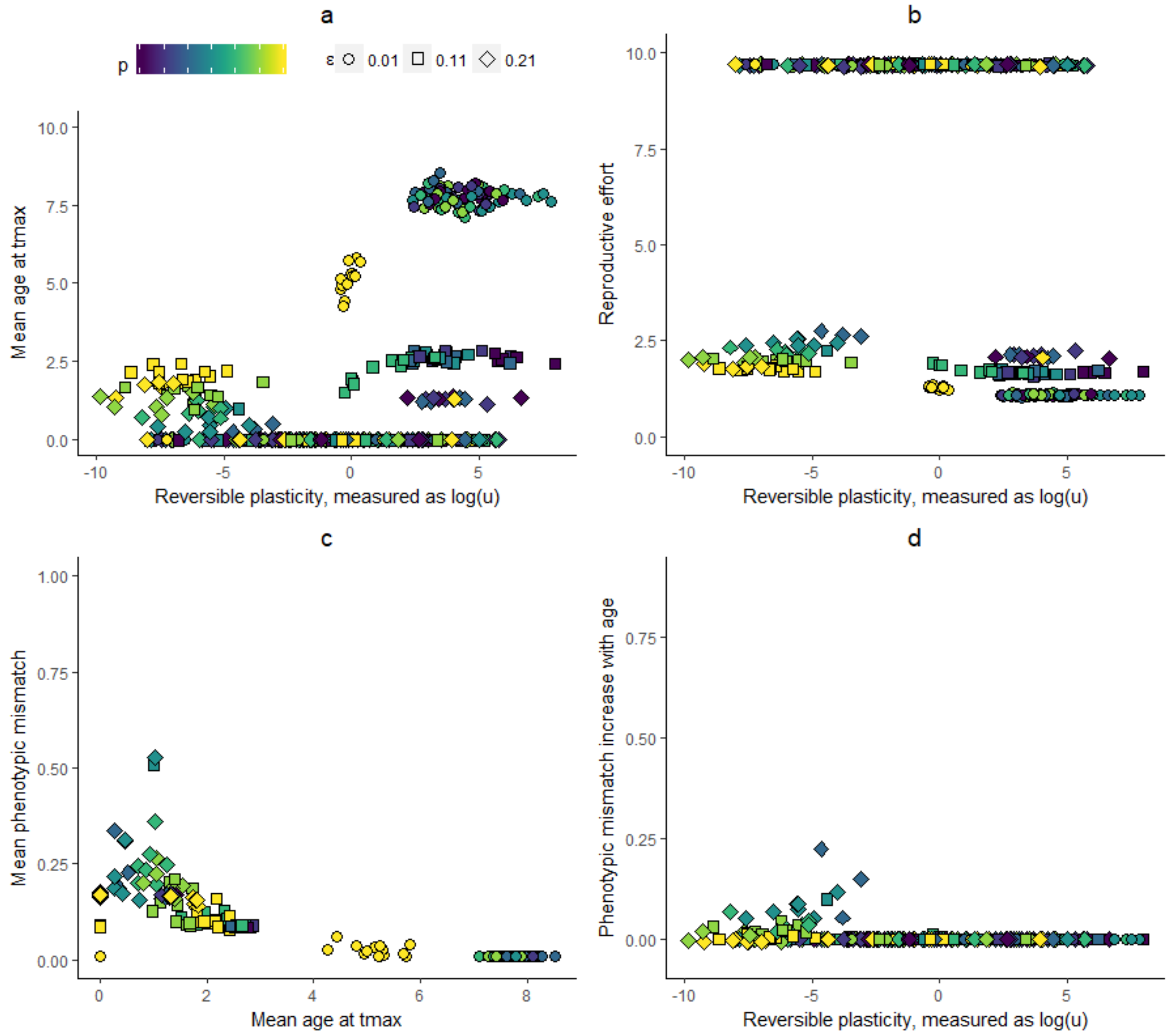

Supplementary Figure 6. Relationships between populations mean trait values at the end of all simulations with high costs of reproduction (ρ=0.05). A) Mean age of individuals — a measure of lifespan — measured at the end of the simulation (t_{max}); long lifespan is only found at high plasticity levels. **B)** Mean evolved reproductive effort (gene value) covaries negatively with plasticity, measured as updating effort, *u*. **C)** Mean population-wide mismatch against mean age of individuals measured at t_{max}. **D)** The regression slope between individual mismatch and individual age, measured at t_{max}, with positive values indicating that older individuals are more mismatched to the current environment. Frequent enough plasticity can prevent this type of senescence. Symbol shapes indicate updating error (ε); colour (from dark blue to yellow) indicates increasing environmental autocorrelation (*p*) ranging from 0.4 to 0.9.

Supplementary Note 3: Phenotypic mismatch affects adult mortality only

In this version of the model, reproductive success is not affected by phenotypic mismatch to the environment; so that: $c_{i,t} = \min(10, r_i) * (1 - \kappa * S_{i,t})$. Only adult mortality is lower with larger mismatch. The results show very similar patterns as in the main model. In the less autocorrelated environments, higher updating error affects more populations to be less reversibly plastic, these also become more phenotypically mismatched and this mismatch increase with age.

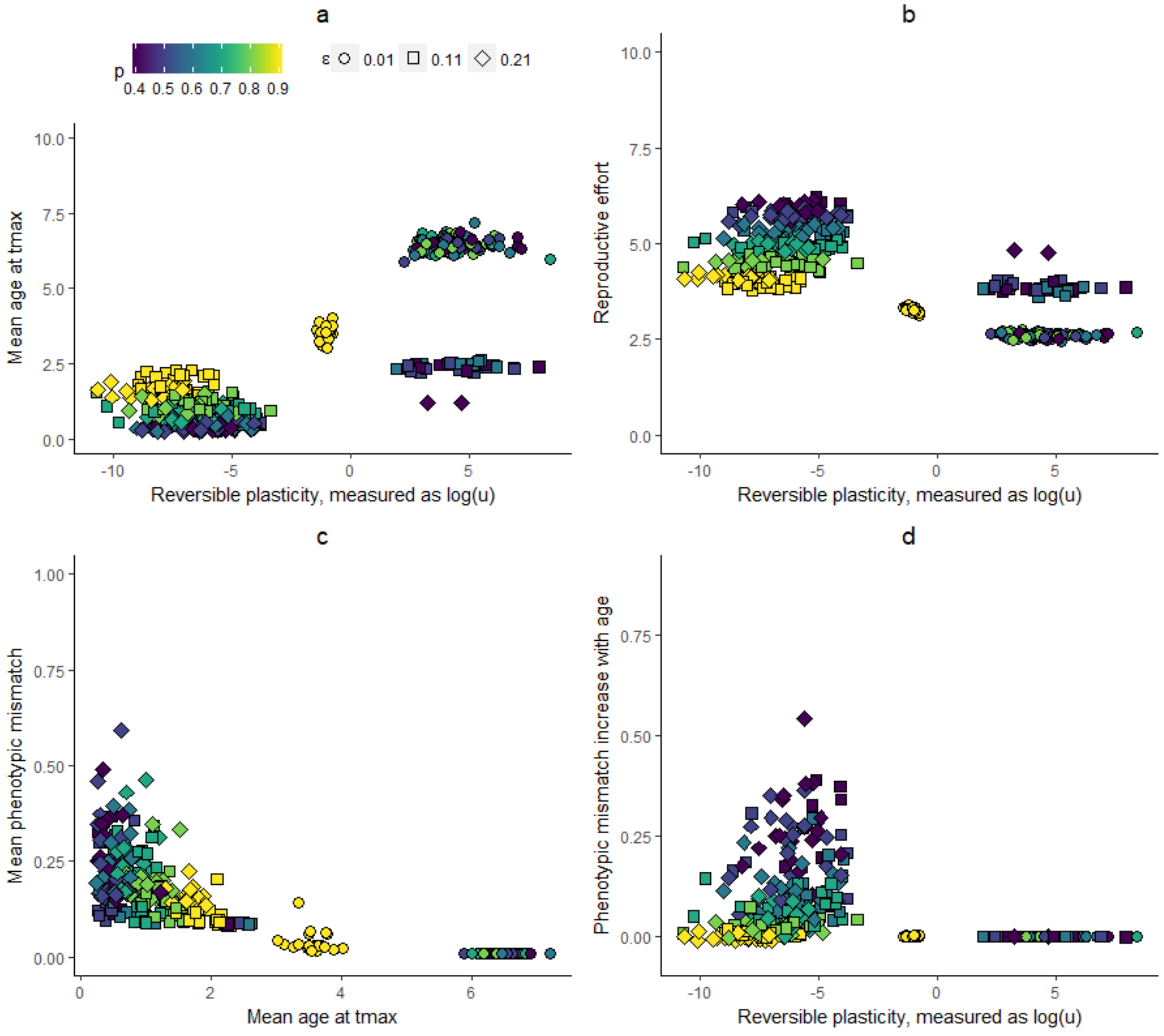

Supplementary Figure 7. Relationships between populations mean trait values at the end of all simulations with phenotypic mismatch affecting adult mortality only. A) Mean age of individuals — a measure of lifespan — measured at the end of the simulation (t_{max}); long lifespan is only found at high plasticity levels. **B)** Mean evolved reproductive effort (gene value) covaries negatively with plasticity, measured as updating effort, *u*. **C)** Mean population-wide mismatch against mean age of individuals measured at t_{max}. **D)** The regression slope between individual mismatch and individual age, measured at t_{max}, with positive values indicating that older individuals are more mismatched to the current environment. Frequent enough plasticity can prevent this type of senescence. Symbol shapes indicate updating error (epsilon); colour (from dark blue to yellow) indicates increasing environmental autocorrelation (*p*) ranging from 0.4 to 0.9.

SUPPLEMENTARY NOTE 4: PHENOTYPIC MISMATCH AFFECT FECUNDITY ONLY

If we let phenotypic mismatch to the environment not affect the adult mortality rate at all: $P(mortality) = \alpha_0 + \kappa\, r_i^2$, but only the fecundity of individuals we do see a change in the results. We now find an opposite and much weaker pattern between reversible plasticity and reproductive effort, and no relationship between reversible plasticity and lifespan. Environmental autocorrelation is now the main factor affecting how much an individual should sample its environment and change its phenotype accordingly. In this scenario, it seems that the benefits of reversible plasticity is only outweighing the costs for those individuals that have a high reproductive effort (but note that variation in reproductive effort and lifespan is quite small). In this scenario trade offs get decoupled, reproductive effort trades off with mortality, while both cost of plasticity and phenotypic mismatch reduces actual reproduction, but independent of the effect of reproductive effort. It is then not surprising that reversible plasticity, *u,* is not related to lifespan, such as in the main model.

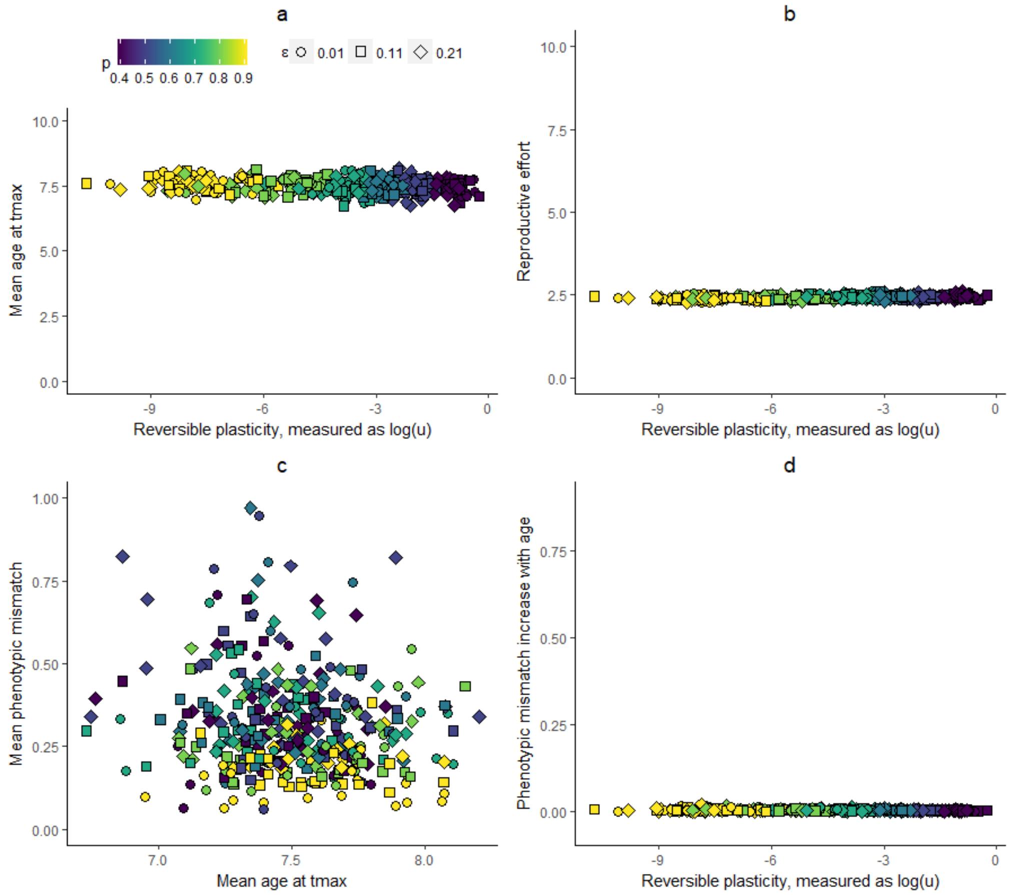

Supplementary Figure 8. Relationships between populations mean trait values at the end of all simulations with phenotypic mismatch affecting adult mortality only. A) Mean age of individuals — a measure of lifespan — measured at the end of the simulation (t_{max}); long lifespan is only found at high plasticity levels. **B)** Mean evolved reproductive effort (gene value) covaries negatively with plasticity, measured as updating effort, *u*. **C)** Mean population-wide mismatch against mean age of individuals measured at t_{max}. **D)** The regression slope between individual mismatch and individual age, measured at t_{max}, with positive values indicating that older individuals are more mismatched to the current environment. Frequent enough plasticity can prevent this type of senescence. Symbol shapes indicate updating error (ε); colour (from dark blue to yellow) indicates increasing environmental autocorrelation (*p*) ranging from 0.4 to 0.9.

SUPPLEMENTARY NOTE 5: ARMA MODELLED ENVIRONMENT

In the main model changing p changes both environmental autocorrelation and total environmental variance. We therefore model the environmental fluctuations using an ARMA model that allow us to have the same total environmental variance but changing the correlation between environments at time t and t+1. Taking into account that there is a correlation between the current environment (E_t) and that in both the previous time step (E_{t-1}) and the time step before that (E_{t-2}), so that the environment varies according to the following function:

$$E_t = \varphi_1 E_{t-1} + \varphi_2 E_{t-2} + Noise_t - \theta\, Noise_{t-1}.$$

Noise is normally distributed with mean 0 and variance σ. Parameters used to get the different environmental autocorrelations are listed in the table below, and environmental variance is ≈ 0.2

Supplementary Table 1. Parameter values and environmental autocorrelations for the ARMA environment.

**p=corr(E_t ,E_{t-1})**	**φ_1**	**φ_2**	**σ**	**θ**
0.17	0.20	-0.05	0.45	0.01
0.45	0.50	-0.10	0.40	0.01
0.68	0.75	-0.05	0.32	0.05
0.81	0.99	-0.01	0.10	0.10
0.96	0.99	-0.01	0.11	0.25

We find that results are still very similar to the results from the main model (Supplementary Figure 9), and changes to the cost of updating has the same effect in this version of the model as in the main version (Compare Supplementary figures 4 and 5 to 11 and 10).

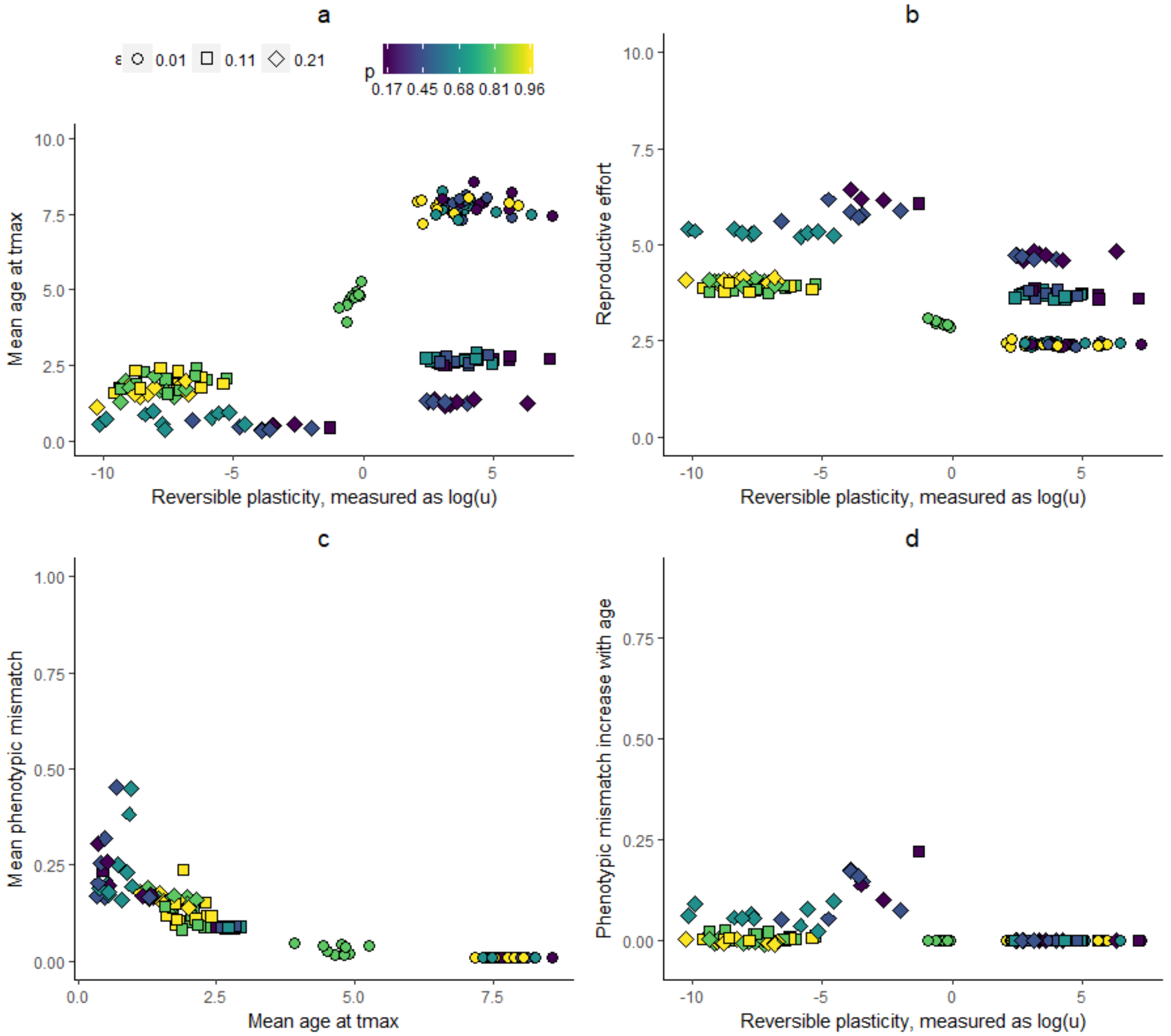

Supplementary Figure 9. Relationships between populations mean trait values at the end of all simulations with ARMA environment. A) Mean age of individuals — a measure of lifespan — measured at the end of the simulation (t_{max}); long lifespan is only found at high plasticity levels. **B)** Mean evolved reproductive effort (gene value) covaries negatively with plasticity, measured as updating effort, *u*. **C)** Mean population-wide mismatch against mean age of individuals measured at t_{max}. **D)** The regression slope between individual mismatch and individual age, measured at t_{max}, with positive values indicating that older individuals are more mismatched to the current environment. Frequent enough plasticity can prevent this type of senescence. Symbol shapes indicate updating error (ε); colour (from dark blue to yellow) indicates increasing environmental autocorrelation (*p*) values given in Supplementary Table 1 with dark blue indicating low autocorrelation to yellow indicating high autocorrelation.

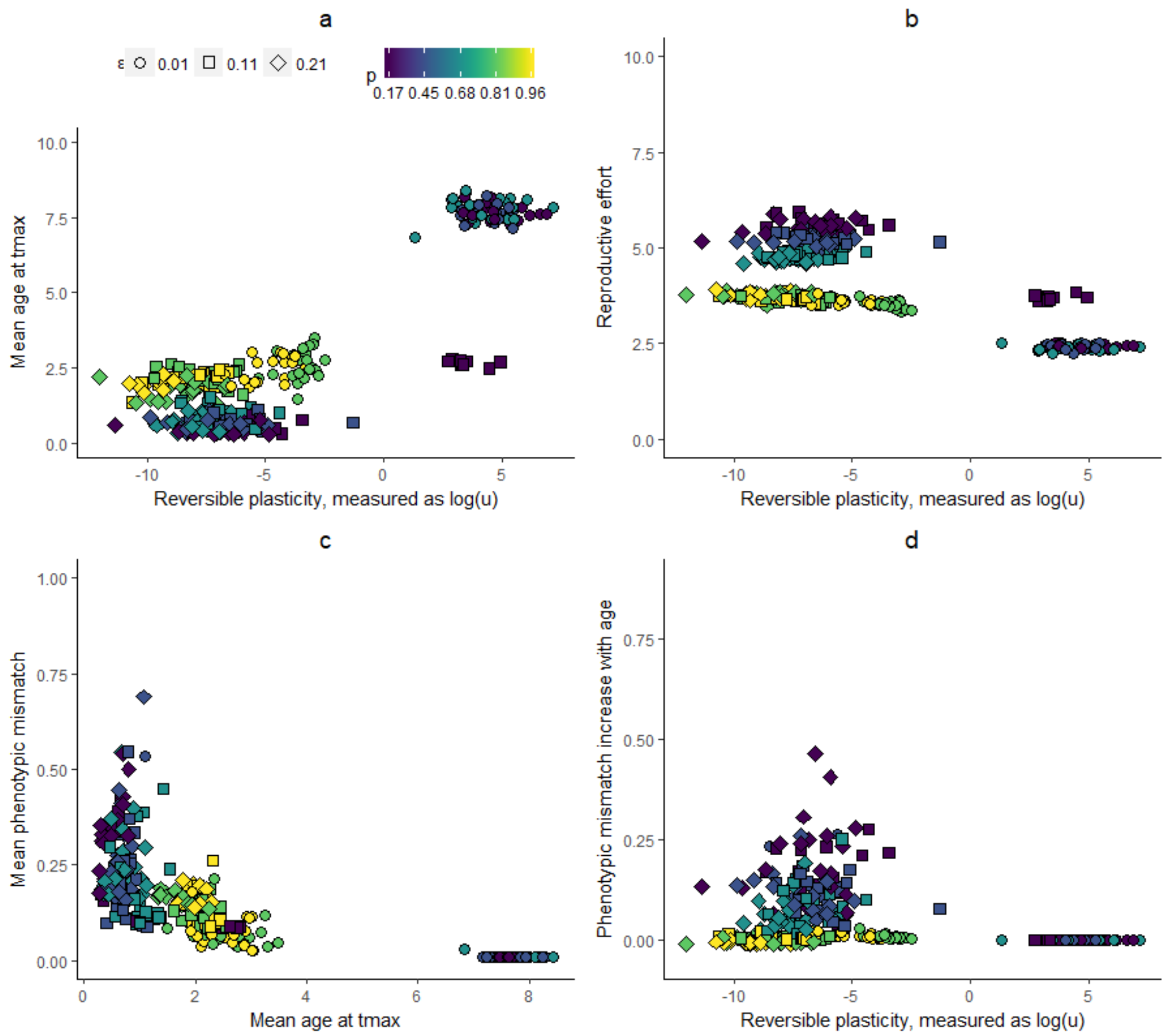

Supplementary Figure 10. Relationships between populations mean trait values at the end of all simulations with ARMA environment and high cost of updating (*κ*=0.6). A) Mean age of individuals — a measure of lifespan — measured at the end of the simulation (t_{max}); long lifespan is only found at high plasticity levels. **B)** Mean evolved reproductive effort (gene value) covaries negatively with plasticity, measured as updating effort, *u*. **C)** Mean population-wide mismatch against mean age of individuals measured at t_{max}. **D)** The regression slope between individual mismatch and individual age, measured at t_{max}, with positive values indicating that older individuals are more mismatched to the current environment. Frequent enough plasticity can prevent this type of senescence. Symbol shapes indicate updating error (ε); colour (from dark blue to yellow) indicates increasing environmental autocorrelation (*p*) values given in Supplementary Table 1 with dark blue indicating low autocorrelation to yellow indicating high autocorrelation.

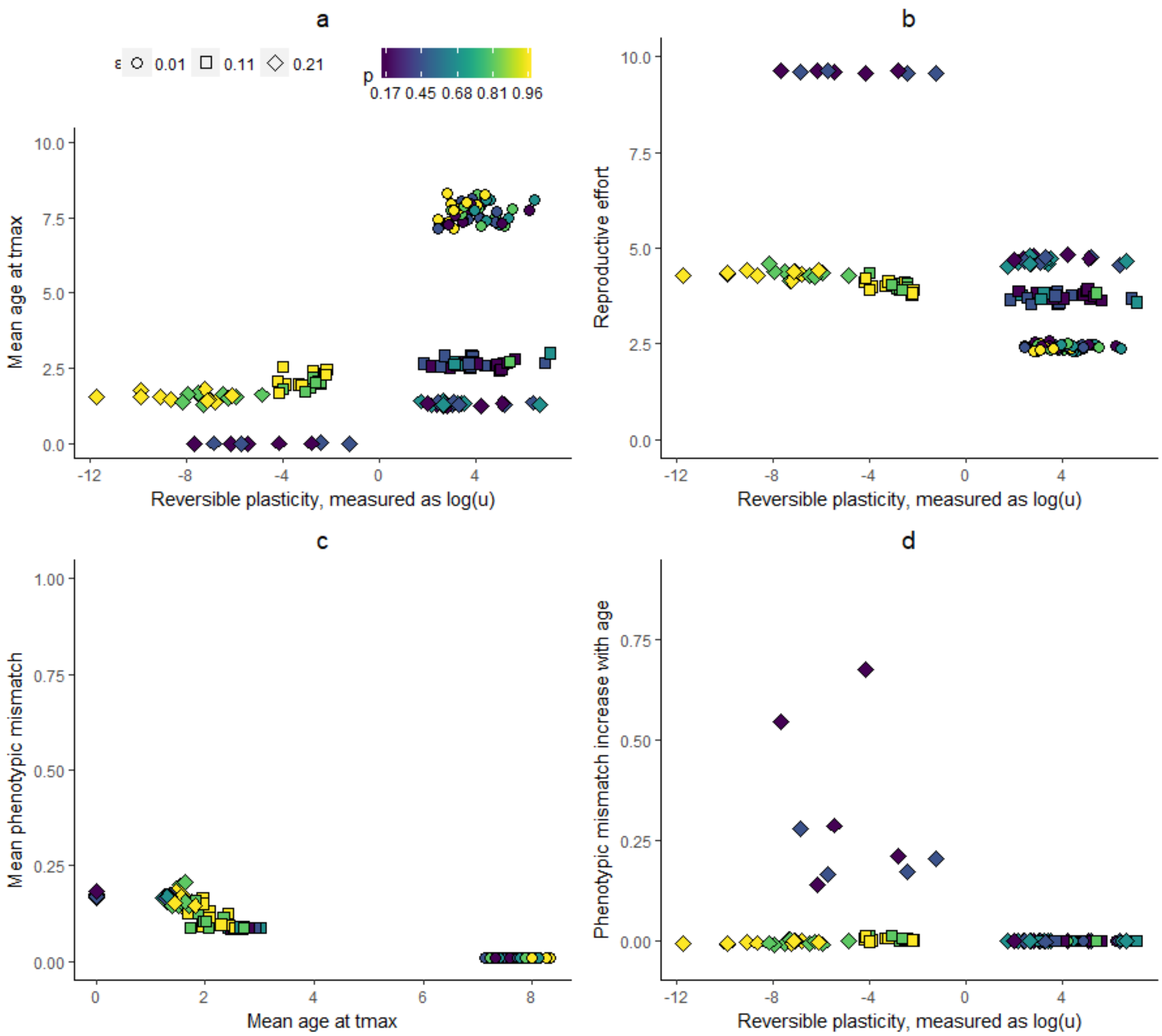

Supplementary Figure 11. Relationships between populations mean trait values at the end of all simulations with ARMA environment and low cost of updating (*κ*=0.2). A) Mean age of individuals — a measure of lifespan — measured at the end of the simulation (t_{max}); long lifespan is only found at high plasticity levels. **B)** Mean evolved reproductive effort (gene value) covaries negatively with plasticity, measured as updating effort, *u*. **C)** Mean population-wide mismatch against mean age of individuals measured at t_{max}. **D)** The regression slope between individual mismatch and individual age, measured at t_{max}, with positive values indicating that older individuals are more mismatched to the current environment. Frequent enough plasticity can prevent this type of senescence. Symbol shapes indicate updating error (ε); colour (from dark blue to yellow) indicates increasing environmental autocorrelation (*p*) values given in Supplementary Table 1 with dark blue indicating low autocorrelation to yellow indicating high autocorrelation.
